# A Single Amino Acid Substitution Prevents Recognition of a Dominant Human Aquaporin-4 Determinant in the Context of HLA-DRB1*03:*01* by a Murine TCR

**DOI:** 10.1371/journal.pone.0152720

**Published:** 2016-04-07

**Authors:** Benjamine Arellano, Rehana Hussain, William A. Miller-Little, Emily Herndon, Doris Lambracht-Washington, Todd N. Eagar, Robert Lewis, Don Healey, Steven Vernino, Benjamin M. Greenberg, Olaf Stüve

**Affiliations:** 1 Department of Neurology and Neurotherapeutics, University of Texas Southwestern Medical Center at Dallas, Dallas, TX, United States of America; 2 Department of Pathology, University of Texas Southwestern Medical Center at Dallas, Dallas, TX, United States of America; 3 Histocompatibility and Transplant Immunology, Department of Pathology and Genomic Medicine, The Methodist Hospital Physician Organization, Houston, TX, United States of America; 4 Opexa Therapeutics, The Woodlands, TX, United States of America; 5 Neurology Section, VA North Texas Health Care System, Medical Service, Dallas, TX, United States of America; 6 Department of Neurology, Klinikum rechts der Isar, Technische Universität München, Munich, Germany; Medical University Vienna, Center for Brain Research, AUSTRIA

## Abstract

**Background:**

Aquaporin 4 (AQP4) is considered a putative autoantigen in patients with Neuromyelitis optica (NMO), an autoinflammatory disorder of the central nervous system (CNS). HLA haplotype analyses of patients with NMO suggest a positive association with HLA-DRB1* *03*:*01*. We previously showed that the human (h) AQP4 peptide 281–300 is the dominant immunogenic determinant of hAQP4 in the context of HLA-DRB1*03:*01*. This immunogenic peptide stimulates a strong Th_1_ and Th_17_ immune response. AQP4_281-300_-specific encephalitogenic CD4^+^ T cells should initiate CNS inflammation that results in a clinical phenotype in HLA-DRB1*03:*01* transgenic mice.

**Methods:**

Controlled study with humanized experimental animals. HLA-DRB1*03:*01* transgenic mice were immunized with hAQP4_281-300_, or whole-length hAQP4 protein emulsified in complete Freund’s adjuvant. Humoral immune responses to both antigens were assessed longitudinally. *In vivo* T cell frequencies were assessed by tetramer staining. Mice were followed clinically, and the anterior visual pathway was tested by pupillometry. CNS tissue was examined histologically post-mortem. Flow cytometry was utilized for MHC binding assays and to immunophenotype T cells, and T cell frequencies were determined by ELISpot assay.

**Results:**

Immunization with hAQP4_281-300_ resulted in an *in vivo* expansion of antigen-specific CD4^+^ T cells, and an immunoglobulin isotype switch. HLA-DRB1*03:*01* TG mice actively immunized with hAQP4_281-300_, or with whole-length hAQP4 protein were resistant to developing a neurological disease that resembles NMO. Experimental mice show no histological evidence of CNS inflammation, nor change in pupillary responses. Subsequent analysis reveals that a single amino acid substitution from aspartic acid in hAQP4 to glutamic acid in murine (m)AQP4 at position 290 prevents the recognition of hAQP4_281-300_ by the murine T cell receptor (TCR).

**Conclusion:**

Induction of a CNS inflammatory autoimmune disorder by active immunization of HLA-DRB1*03:*01* TG mice with human hAQP4_281-300_ will be complex due to a single amino acid substitution. The pathogenic role of T cells in this disorder remains critical despite these observations.

## Introduction

Neuromyelitis optica (NMO) is a demyelinating inflammatory disorder of the central nervous system (CNS) that is clinically and pathologically defined as the co-occurrence of optic neuritis and myelitis [1, 2]. Aquaporin (AQP)4 is considered a potential autoantigen in patients with NMO after an autoantibody, designated NMO-IgG, that binds to human (h) AQP4 was detected in the serum of the vast majority of patients with NMO [3, 4]. The presence of the NMO-IgG has led many neurologist and neuroimmunologists to believe that NMO may be a primarily B cell-mediated disease.

However, there is evidence to suggest a cellular immune response in NMO during disease initiation or perpetuation [5, 6]. HLA haplotype analyses of patients with NMO suggest a positive association with HLA-DRB1* *03*:*01* (DR17) [7, 8] [9], a gene that codes for a major histocompatibility class (MHC) II molecule that presents linear antigens to CD4^+^ T cells [10]. Also, NMO-IgG is undetectable in a substantial number of patients with NMO [3]. A NMO-IgG, antibody isotype switch from IgM to IgG could not occur without CD4^+^ T cell involvement [11, 12], which are abundantly present in NMO lesions [13]. B cell–depleting therapies are not consistently beneficial in patients with NMO [14–16]. Finally, transfer of AQP4-reactive T cells into wild-type mice and rats results in neurological deficits and CNS inflammation [17, 18].

Other investigators have identified immunogenic linear determinants in various rodent species [5, 6, 19–21]. We have previously shown that human AQP4 peptide 281–330 (hAQP4_281-300_) is the dominant immunogenic determinant of hAQP4 in the context of HLA-DRB1*03:01 [21]. Characterizing the encephalitogenic role of these AQP4 specific T helper cells will bring to light the role of the cellular immune response in the initiation and progression of the NMO clinical disease phenotype.

In this study we intended to establish a T cell-mediated animal model of NMO in the context of HLA-DRB1*03:*01*, utilizing hAQP4_281-300_ as the dominant hAQP4 determinant in that MHC II haplotype. Induction of an autoimmune disorder resembling experimental autoimmune encephalomyelitis (EAE)[22] was attempted by active immunization and adoptive transfer. Clinical disease activity, CNS tissue inflammation, and changes in pupillary reflexes were assessed. Alanine scanning of AQP4_281-300_ was performed to test recognition by mouse T cell receptors (TCRs) and HLA-DRB1*03:*01*.

We were unable to induce clinical EAE, CNS inflammation, or altered pupillary responses. Disease resistance is the result of a single amino acid substitution from aspartic acid in hAQP4 to glutamic acid in murine (m)AQP4 at position 290 prevents the recognition of hAQP4_281-300_ by the murine T cell receptor (TCR).

## Results

### Immunization with human (h)AQP4_281-300_ leads to an expansion of antigen-specific CD4^+^ T cells in vivo

**Fig 1:**
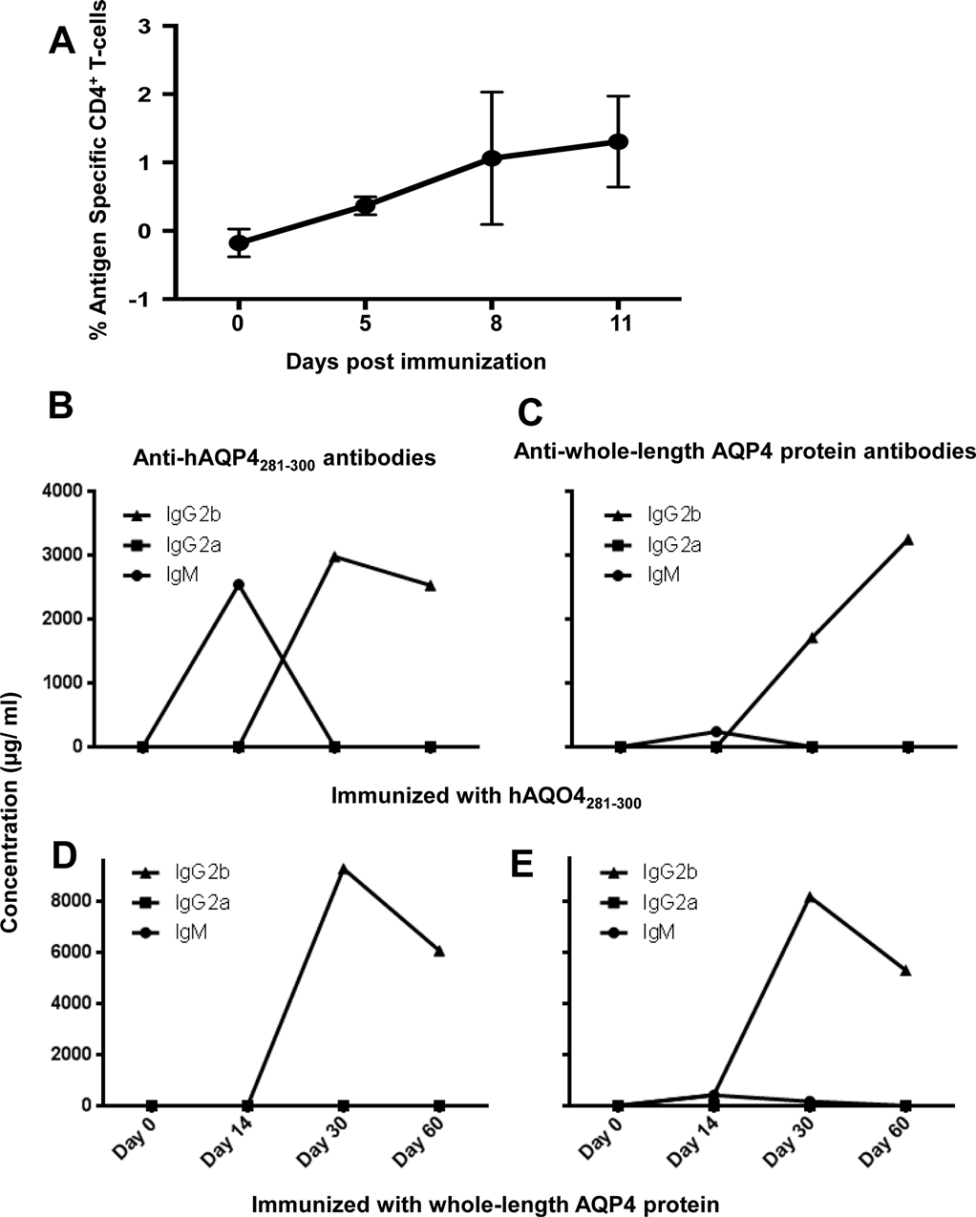
Immunization with human (h)AQP4_281-300_ leads to an expansion of antigen-specific CD4^+^ T cells *in vivo*, and an Ig isotype switch in HLA-DRB1*03:*01* transgenic mice. (A) Following immunization with human (h)AQP4_281-300_, an expansion of antigen-specific CD4^+^ T helper cells was detected by tetramer staining of lymph node cells. The fluorescent signal of HLA-DRB1*03:*01*-loaded tetramers minus the fluorescent signal of empty HLA-DRB1*03:*01* tetramers is shown. CD4^+^ T helper cells provide soluble mediators that drive B cell differentiation immunoglobulin (Ig) class switching. To determine whether hAQP4_281-300_-reactive CD4^+^ T cells are capable of causing IgM to IgG isotype switching in HLA-DRB1*03:*01* transgenic mice, the concentration of Ig against hAQP4_281-300_, mAQP4284-299, or with whole-length hAQP4 protein in serum of immunized mice was quantified longitudinally. Since the NMO-IgG is a human IgG1 isotype, both, the murine IgG2a and IgG2b isotype were examined as they have similar properties with regard to complement binding and the Fcγ receptor. A switch from IgM to IgG2b was detected in mice immunized with hAQP4_281-300_ peptide with regard to (B) antibody responses against hAQP4_281-300_ and (C) whole-length AQP4 protein. An Ig isotype switch from IgM to IgG2b was also detectable in mice immunized with whole-length AQP4 protein with regard to (D) antibody responses against hAQP4_281-300_ and (E) whole-length AQP4 protein.

Following immunization with human (h)AQP4_281-300_ an expansion of antigen-specific CD4^+^ T helper cells was detected by tetramer staining of lymph node cells (**Fig 1A**).

### Immunization with human (h)AQP4_281-300_ leads to an Ig isotype switch in HLA-DRB1*03:01 transgenic mice

CD4^+^ T helper cells provide soluble mediators that drive B cell differentiation immunoglobulin (Ig) class switching. To determine whether hAQP4_281-300_-reactive CD4^+^ T cells are capable of causing IgM to IgG isotype switching in HLA-DRB1*03:*01* transgenic mice, the concentration of Ig against hAQP4_281-300_, mAQP4284-299, or with whole-length hAQP4 protein in serum of immunized mice was quantified longitudinally. Since the NMO-IgG is a human IgG1 isotype, both, the murine IgG2a and IgG2b isotype were examined as they have similar properties with regard to complement binding and the Fcγ receptor. A switch from IgM to IgG2b was detected in mice immunized with hAQP4_281-300_ peptide with regard to antibody responses against hAQP4_281-300_ (**Fig 1B**), and whole-length AQP4 protein (**Fig 1C**). An Ig isotype switch from IgM to IgG2b was also detectable in mice immunized with whole-length AQP4 protein with regard to antibody responses against hAQP4_281-300_ (**Fig 1D**), and whole-length AQP4 protein (**Fig 1E**). Thus, B cells of HLA-DRB1*03:*01 transgenic mice* are capable of recognizing hAQP4_281-300_ peptide via the B cell receptor (BCR), and the cellular immune response against hAQP4_281-300_ subsequently drives Ig isotype switching. These data support our previously published data that hAQP4_281-300_ is a dominant determinant in HLA-DRB1*03:01 [21].

### Active immunization with hAQP4 does not lead to clinical disease

**Fig 2:**
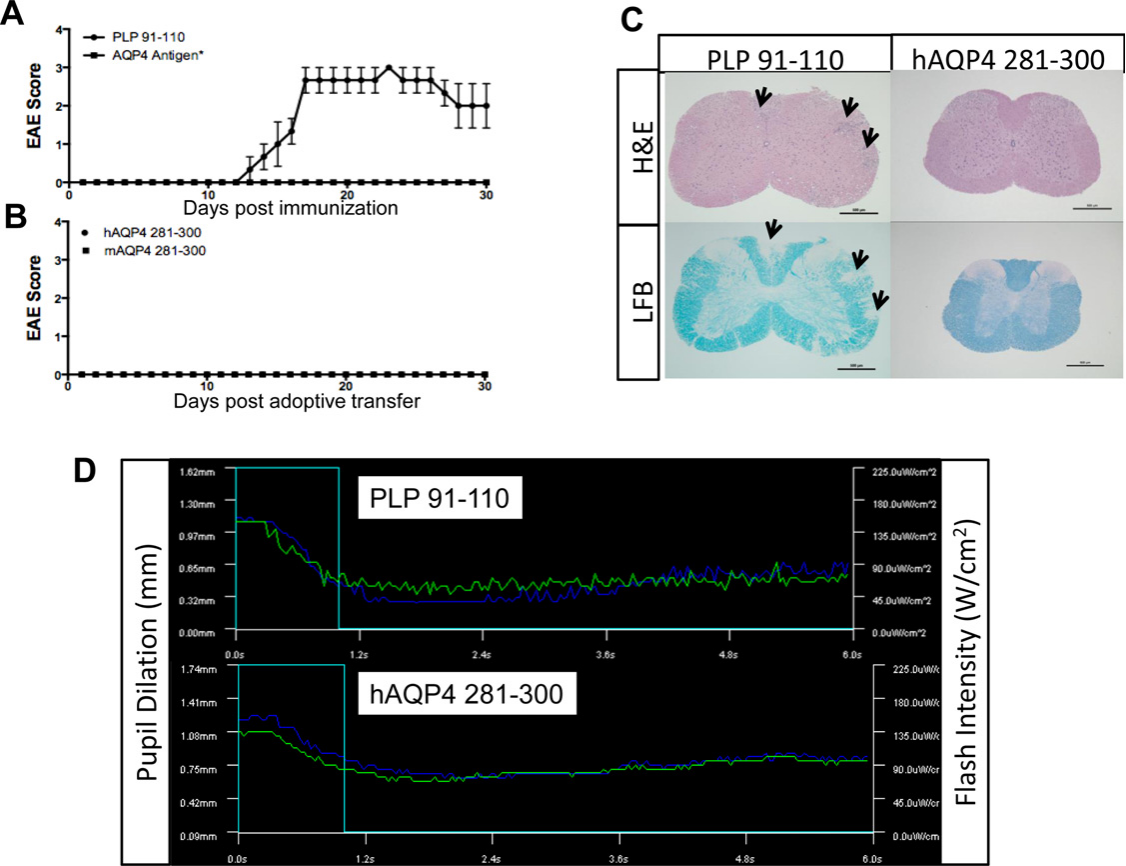
HLA-DRB1*03:*01* transgenic mice are disease resistant to active immunization with human aquaporin 4 (hAQP4), and adoptive transfer of hAQP4-specific T cells. (A) HLA-DRB1*03:*01* mice were actively immunized with proteolipid protein (PLP)_91-110_ (100 μg/100 μl/mouse; positive control [25]), or varying AQP4 antigens*(whole-length hAQP4 protein, hAQP4_281-300_, murine (m)AQP4_281-300_, hAQP4_281-300_ with a Quil-A Incomplete Freund Adjuvant (IFA) booster on day 14 post-immunization, mAQP4_281-300_ with a Quil-A IFA booster on day 14 post immunization, and hAQP4_281-300_ plus mAQP4_281-300_) emulsified in Complete Freund Adjuvant (CFA). Immunization with a positive control proteolipid protein (PLP)_91-110_, a dominant encephalitogenic determinant in HLA-DRB1*03:*01* led to typical EAE. (B) Lymph node cells taken from HLA-DRB1*03:*01* mice immunized with hAQP4_281-300_ or mAQP4_281-300_ were restimulated for three days and passively transferred into HLA-DRB1*03:*01* mice. None of these experimental approaches resulted in clinical disease. (C) Paraffin sections were stained with haematoxlin eosin (H&E) and luxol fast blue (LFB). Representative sections of the spinal cords from PLP_91-110_ and hAQP4_281-300_ immunized mice are shown. On histopathological examination there were no visible signs of cellular infiltration, inflammation, or demyelination within the brain and spinal cord in any experimental paradigms other than in active immunization with PLP_91-110_, the dominant encephalitogenic determinant in HLA-DRB1*03:*01* that led to typical EAE (spinal cord shown; inflammatory infiltrates and areas of demyelination are indicated by black arrows). (D) Fifteen days post immunization of HLA-DRB1*03:*01* transgenic mice with PLP_91-110_ or hAQP4_281-300_, pupillary reflex was measured via a mouse pupillometry. Mice actively immunized with hAQP4_281-300_ and the control antigen PLP_91-110_ did not show altered pupillary responses.

We first examined whether active immunization of HLA-DRB1*03:*01* transgenic [23] mice with hAQP4 results in clinical disease. A multitude of experimental procedures and conditions were tested to examine the encephalitogenic potential of hAQP4 peptides using the transgenic mice. Previously, our laboratory determined that hAQP4_281-300_ was capable of generating a strong Th_1_ and Th_17_ immune response as measured by IFNγ and IL-17 ELISpot assay [21]. We performed active immunization with whole-length hAQP4 protein, hAQP4_281-300_, or mAQP4_281-300_ in an attempt to generate an animal model of NMO. Experimental animals also received intraperitoneal (i.p.) injections of pertussis toxin (Ptx) on the day of immunization and two days post immunization [24]. This approach resulted in no observable clinical paralysis commonly seen in EAE models (**Fig 2A**). Immunization with a positive control proteolipid protein (PLP)_91-110_, the dominant encephalitogenic determinant in HLA-DRB1*03:*01* [25] led to typical EAE (**Fig 2A**). All EAE experiments were terminated at day 30. None of the experimental animals immunized with PLP_91-110_ that developed EAE died prematurely.

Subsequently, alternative methods to generate a T cell-mediated NMO model were employed. Some CNS autoimmune disease animal models require weekly booster immunization to generate disease phenotypes [26]. The rationale for this approach is to increase the encephalitogenic potential of hAQP4_281-300_ by overcoming mechanisms of peripheral tolerance. In other experiments, additional booster immunization were given at day fourteen with adjuvants other than CFA, including QuilA and incomplete Freund’s adjuvant (IFA)_._ Again, no clinical disease was observed (**Fig 2A**).

Mice were also co-immunized mice with both hAQP4_281–300_ and mAQP4_281-300_. Again, there was complete disease resistance (**Fig 2A**).

### Adoptive transfer of hAQP4281-300-specific CD4^+^ T cells does not lead to clinical disease

In the adoptive transfer EAE model myelin-reactive activated CD4^+^ T cells are transferred into a naïve recipient. This model has some propensities that are very different from actively-induced EAE: [27] The potential effects of adjuvant and pertussis toxin on the innate immune system are eliminated, and [27] *in vitro* re-activated donor T cells are less dependent on reactivation within the recipient CNS [28]. This model was specifically developed to test the role of antigen-specific donor T cells in EAE pathogenesis [29]. Since Th_1_ and Th_17_ cells have been shown in the EAE model to be capable of causing disease when passively transferred, we next examined whether hAQP4_281-300_-specific T cells could cause disease via adoptive transfer. No disease phenotype was detected (**Fig 2B**). These results indicate that reactivation of hAQP4_281-300_–specific or mAQP4_281-300_–specific CD4^+^ Th_1_ cells does not occur in the CNS of HLA-DRB1*03:*01* recipient mice.

On histopathological examination there were no visible signs of cellular infiltration, inflammation, or demyelination within the brain and spinal cord in any experimental paradigms other than in active immunization with PLP_91-110_, the dominant encephalitogenic determinant in HLA-DRB1*03:*01* that led to typical EAE (spinal cord shown in **Fig 2C**; inflammatory infiltrates and areas of demyelination are indicated by black arrows). The absence of any functional deficits was further corroborated through measuring of the pupillary reflex by murine pupillometry on day 15 post immunization (**Fig 2D**). Mice did not show altered pupillary responses, further substantiating our previous findings that no functional or structural damage had occurred within the optic nerve.

The outcomes of these experiments suggested that mAQP4_281-300_ cannot be recognized in the context of HLA-DRB1*03:*01*, or that hAQP_281-300_ cannot be recognized by B.10 TCR.

### Single Amino Acid difference leads to blocking of hAQP4-mediated T cell proliferation and differentiation

**Fig 3:**
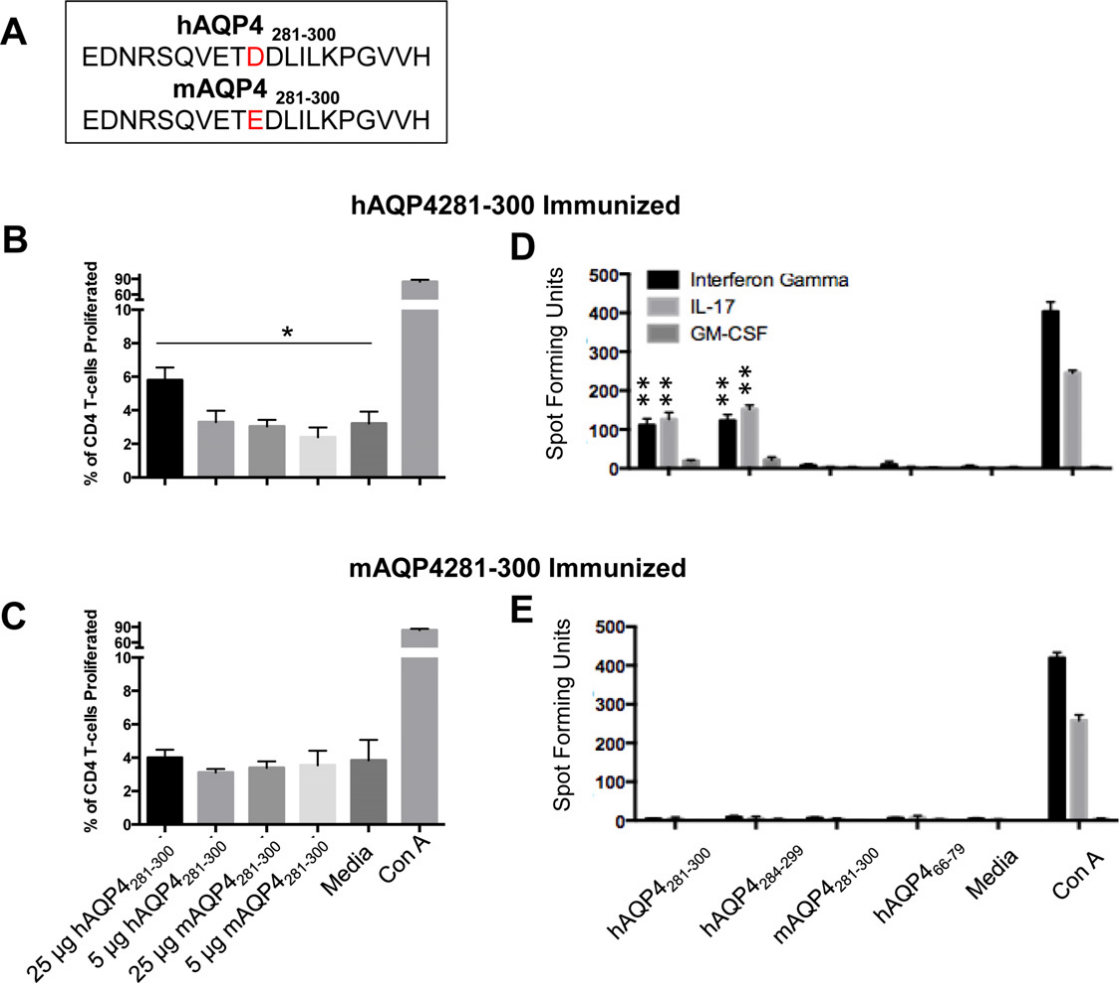
Human (h)AQP4_281-300_-specific T cells do not cross-react with murine (m) AQP4_281-300._ (A) There is that a single amino acid substitution from aspartic acid in hAQP4 to glutamic acid in murine (m)AQP4 at position 290. (B) In lymph node cells of HLA-DRB1*03:*01* mice immunized with hAQP4_281-300_ there was a significant proliferation of CD4^+^ T cells when hAQP4_281-300_ was used as the recall antigen (* = P-value = 0.01). Only a higher recall antigen dose of 25 μg/ml resulted in a significant increase in proliferation, whereas as a dose of 5 μg/ml did not. (C) There was no proliferative response to mAQP4_281-300_ at either dose_._ (D) There is a significantly increased frequency of IFNγ and IL-17 producing lymph nodes cells from HLA-DRB1*03:*01* mice immunized with hAQP4_281-300_ by ELISpot assay when hAQP4_281-300_, and hAQP4_281-299_ are used as recall antigens. However, we were unable to detect antigen specific IFNγ and IL-17 producing lymph nodes cells when mAQP4_281-300_, or the negative control hAQP4_66-79_ were used as recall antigens (** = P-value < 0.01). (E) IFNγ and IL-17 producing lymph nodes cells from HLA-DRB1*03:*01* mice immunized with mAQP4_281-300_ were undetectable with any of the recall antigens.

Within the immunogenic hAQP4_281-300_, there is a single amino acid mutation at position 290 between the human peptide and the mouse analog: An aspartic acid (D) in the human peptide to glutamic acid (E) in the mouse (**Fig 3A**). Both are negatively charged acidic amino acids that contain a carboxylic acid at the end of their side-chains. The difference between the two amino acids is an additional methyl group in the side chain of glutamic acid.

In lymph node cells of HLA-DRB1*03:*01* mice immunized with hAQP4_281-300_ there was a significant proliferation of both CD4^+^ T cells when 25 μg/ml of hAQP4_281-300_ was used as the recall antigen (**Fig 3B**). A dose of 5 μg/ml hAQP4_281-300_, or mAQP4_281-300_ did not result in a significant proliferative response (**Fig 3B**). Lymph node cells of HLA-DRB1*03:*01* mice immunized with mAQP4_281-300_ did also not proliferate in response to mAQP4_281-300_, or hAQP4_281-300_ at either dose (**Fig 3C**)_._ An ELISpot assay revealed a significantly increased frequency of IFNγ and IL-17 producing lymph nodes cells from HLA-DRB1*03:*01* mice immunized with hAQP4_281-300_ by ELISpot assay when hAQP4_281-300_ and hAQP4_284-299_ are used as recall antigens (**Fig 3D**). However, we were unable to detect antigen specific IFNγ and IL-17 producing lymph nodes cells when mAQP4_281-300_, or the negative control hAQP4_66-79_ were used as recall antigens. IFNγ and IL-17 producing lymph nodes cells from HLA-DRB1*03:*01* mice immunized with mAQP4_281-300_ were undetectable with any of the recall antigens (**Fig 3E**).

Our observations suggest that the aspartic acid residue plays a critical role in either the presentation of hAQP4_281-300_ in the context of HLA-DRB1*03:*01*, or in the recognition of hAQP4_281-300_ by the B.10 TCR.

### hAQP4_281-300_ and mAQP4_281-300_ binds to the HLA-DRB1*03:01 MHC II molecule

With no detectable cellular immune response against mAQP4_281-300_ in HLA-DRB1*03:*01*, we next examined whether the single amino acid mutation affected the anchoring of the mouse peptide to the HLA-DRB1*03:*01* molecule. To identify critical residues of the AQP4 peptides, alanine-scanning peptides were generated that replaced each amino acid of mAQP4_281-300_ with an alanine to aid in distinguishing anchor residues from contact residues (**Table 1**). Since we previously identified hAQP4_284-299_ to be the immunogenic region within hAQP4_281-299_ [21], the alanine scanning peptides assessed only these residues.

**Table 1:** Human (h)AQP4_284-299_ Alanine Scanning Peptides. The immunogenic region of hAQP4_281-300_, hAQP4_284-299_, was utilized to generate alanine scanning peptides at which each peptide sequence has a single alanine residue mutation.

Peptide	Amino Acid Sequence
Human 284–299	RSQVET**D**DLILKPGVV
Mouse 284–299	RSQVET**E**DLILKPGVV
R284A	**A**SQVETDDLILKPGVV
S285A	R**A**QVETDDLILKPGVV
Q286A	RS**A**VETDDLILKPGVV
V287A	RSQ**A**ETDDLILKPGVV
E288A	RSQV**A**TDDLILKPGVV
T289A	RSQVE**A**DDLILKPGVV
D290A	RSQVET**A**DLILKPGVV
D291A	RSQVETD**A**LILKPGVV
L292A	RSQVETDD**A**ILKPGVV
I293A	RSQVETDDL**A**LKPGVV
L294A	RSQVETDDLI**A**KPGVV
K295A	RSQVETDDLIL**A**PGVV
P296A	RSQVETDDLILK**A**GVV
G297A	RSQVETDDLILKP**A**VV
V298A	RSQVETDDLILKPG**A**V
V299A	RSQVETDDLILKPGV**A**

**Fig 4:**
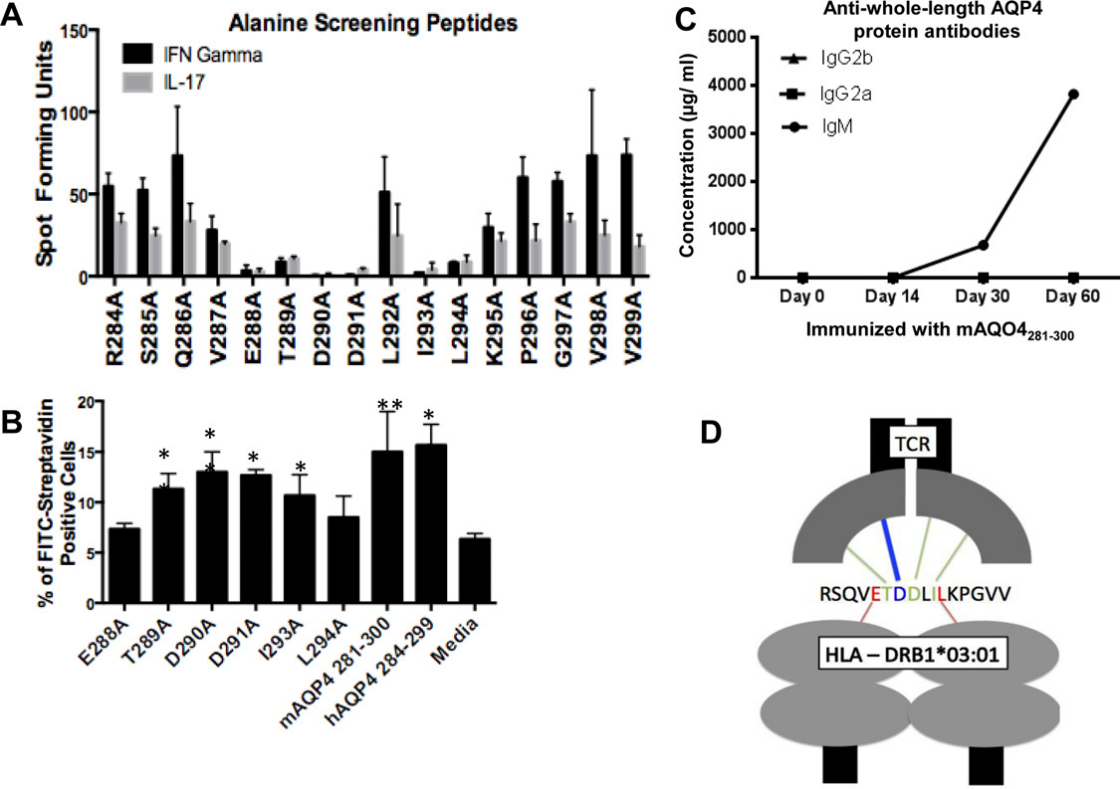
Identification of critical residues of human (h)AQP4_281-300_ for presentation in the context of HLA-DRB1*03:*01* and recognition by the B.10 T cell receptor (TCR). (A) First, the ability of hAQP4_281-300_-reactive lymph node cells to recognize the alanine screening peptides was determined by ELISpot. 5.0x10^5^ cells/well lymph node cells taken ten days post immunization of HLA-DRB1*03:*01* transgenic mice with hAQP4_281-300_ were restimulated with hAQP4 alanine scanning peptides (2 5 μg/mL) for 48 hours in IFNγ and IL-17 ELISpot plates (* = P-value < 0.05 and ** = P-value < 0.01). (B) Alanine screening peptides that not result in an increased frequency of IFNγ and IL-17 secreting lymph node cells were identified as the key residue peptides, and were subsequently tested in a MHC binding assay. Splenocytes taken from HLA-DRB1*03:*01* transgenic mice were incubated for 12 hours in the presence of biotinylated hAQP4 alanine scanning peptides. Post incubation, cells were stained utilizing FITC-Avidin, and antigen positive cells were quantified by flow cytometry (* = P-value < 0.05 and ** = P-value < 0.01). (C) There was no Ig isotype class switch in mice immunized with mAQP4_284-299_ with regard to antibody responses against whole-length AQP4 protein. (D) Critical HLA-DRB1*03:*01* anchor residues, and B.10 TCR contact amino acids are specified. E_288_ and L_294_ are required as HLA-DRB1*03:*01* anchor residues, while T_289_, D_290_, D_291_, and I_293_ are critical B.10 TCR interacting residues.

First, the ability of hAQP4_281-300_-reactive lymph node cells to recognize the alanine screening peptides was determined by ELISpot (**Fig 4A**). Alanine screening peptides that not result in an increased frequency of IFNγ and IL-17 secreting lymph node cells were identified as the key residue peptides. Utilizing a flow-cytometry based MHC II binding assay, we were able to delineate between anchor residues and TCR contact residues. To perform the flow-cytometry based MHC II binding assay, peptides were biotinylated so that when presented in the context of HLA-DRB1*03:*01*, a FITC-avidin would distinguish peptides that were capable of being presented from those that could not. In comparing the percent of FITC-avidin positive cells, amino acids 288E and 294L were identified as the main anchor residues that interact with the HLA-DRB1*03:*01* MHC II molecule (**Fig 4B**). The remaining residues, including the 290D residue that distinguishes mAQP4_281-300_ and hAQP4_281-300_, were not required for binding. As a negative control, alanine scanning peptides were tested in C57BL/6 mice to examine the critical residues for binding to the *H-2b* MHC II molecule. Despite hAQP4_281-300_ being able to be presented on the *H-2b* MHC II molecule, the critical residues were not similar to the critical residues necessary for binding to the HLA-DRB1*03:*01* MHC II molecule. This observation may explain why we were unable to elicit cellular immune response against hAQP4_281-300_ in C67BL/6 mice immunized with this peptide (data not shown).

These data indicate that the mAQP4_281-300_ binds to the HLA-DRB1*03:*01* molecule via the same anchor residues and is thus able to be presented on the MHC II molecule in HLA-DRB1*03:*01* transgenic mice.

### AQP4 residue 290 mediates recognition by the B.10 TCR

With mAQP4_281-300_ being capable of being presented on the HLA-DRB1*03:*01* molecule, we next examined whether the single amino acid mutation inhibited the contact with the hAQP4_281-300_ specific B.10 TCR. Utilizing the alanine-scanning peptides, it was possible to identify critical residues that are required for generating the cellular immune response against the peptide. In performing IFNγ and IL-17 ELISpot assays with lymph node cells restimulated with alanine screening peptides (**Table 1**), E288A, T289A, D290A, D291A, I293A, and L294A were identified as critical amino acids for either the binding of the peptide to HLA-DRB1*03:*01*, or interacting the AQP4_281-300_ specific T cell receptor (TCR). None of the other amino acids are required for either binding to HLA-DRB1*03:*01* molecule, or recognition by the B.10 TCR.

Subsequently, utilizing a MHC binding assay, only the previously identified peptides E288A, T289A, D290A, D291A, I293A, and L294A were screened for their ability to bind to HLA-DRB1*03:*01*. 288E and 294L were identified to be HLA-DRB1*03:*01* anchor residues, and the remaining residues play a critical role in the contact between the peptide and B.10 TCR (**Fig 4B**). These observations suggest that the D to E mutation between hAQP4 and mAQP4 peptides prevent the activation of hAQP4-specific T cells against mAQP4_281-300_.

We did not observe Ig class switching in mice immunized with mAQP4_284-299_ against whole-length AQP4 protein (**Fig 4C**), substantiating our observation that immunization with mAQO4_284-299_ does not drive antigen-driven T cell activation and a T cell proliferative response.

## Discussion

In this investigation, we show that immunization with the immunogenic, hAQP4_281-300_ determinant in HLA-DRB1*03:*01* Tg mice, while leading to an expansion of antigen-specific CD4^+^ T cells, an Ig isotype switch of anti-hAQP4_281-300_ antibodies, and a robust Th_1_ and Th_17_ immune response, does not lead to a clinical disease phenotype via active immunization or passive transfer of hAQP4_281-300_–specific CD4^+^ T cells. We also were unable to detect any evidence of electrophysiological anterior pathway pathology, or any evidence of CNS infiltration in our mouse model. It is conceivable that there may have been inflammatory infiltrates within the meninges of the brain and spinal cord. This was not assessed.

In summary, our data suggest that a single amino acid substitution between hAQP4 and mAQP4 is one possible reason why it will be challenging to establish an animal model of NMO in HLA-DRB1*03:*01* transgenic mice. For induction of CNS autoimmunity, recognition of the cognate antigen by the host immune system is an absolute requirement [24, 28]. Within the pathogenic AQP4_281-300_, the glutamic acid (D) to aspartic acid (E) mutation results in the addition of a methyl group within the side chain of the AQP4_287_ residue in the murine peptide. Despite being the same polarity, the hAQP4_281-300_-specific TCR can differentiate between the human and the mouse peptides. This was corroborated with data showing that the AQP4_287_ residue was important for contact with the B.10 TCR rather than being a MHC II anchor residue. Furthermore, antigen recall with mAQP4_281-300_ in HLA-DRB1*03:*01* mice immunized with mAQP4_281-300_ does not result in proliferation of CD4^+^ T cells. Likely, negative thymic selection for mAQP4_281-300_ specific CD4^+^ T cells occurs in these mice, and prevents active disease induction.

T cell mediated disease models of NMO will be necessary to fully understand the complexity of this disorder. Induction of a CNS inflammatory autoimmune disorder by active immunization of HLA-DRB1*03:*01* TG mice with human hAQP4_281-300_ will be complex due to a single amino acid substitution.

It is also possible that hAQP4_281-300_, the dominant determinant of hAQP4 in HLA-DRB1*03:*01 in vitro*, does not result in generation of encephalitogenic CD4^+^ T cells *in vivo*. This possibility has not conclusively been ruled-out.

The pathogenic role of T cells in this disorder remains critical despite these observations.

## Methods

### Mice

The HLA-DRB1*03:*01* transgenic mice was given to our laboratory by Dr. Chella David and was previously described in *Strauss et al* [30]. Briefly, transgenic mice were generated by co-injection of a HLADRoL genomic fragment and a DRB1*030113 gene fragment into (C57BL/6 x DBA/2) F1 C57BL/6 embryos, and backcrossed to B10 mice [30]. Subsequently, the DRB1*030113 gene was introduced into the class II-negative H2^q-/-^ strain (16) by mating the B10.M-DRBI*0301 line with the B10.MHCII ^/-^ line. C57BL/6 mice were purchased from (The Jackson Laboratories, Bar Harbor, MN). All mice were bred and maintained in a pathogen free mouse colony at the University of Texas Southwestern Medical Center (UTSW) with accordance to the guidelines set forth by the National Institute of Health and our institution. All experiments pertaining to these animals are approved by the UTSW Institutional Animal Care and Use Committee (IACUC).

### Proteins and peptides

Whole-length AQP4 M1 protein was donated by Dr. William Harries of the Membrane Protein Expression Center & Center for Structures of Membrane Proteins Macromolecular Structure Group (UCSF, San Francisco, CA.

The twenty-amino acid-long synthetic hAQP4_281-300_ (EDNRSQVETDDLILKPG VVH), mAQP4_281-300_ (EDNRSQVETEDLILKPGVVH), the hAQP4_281-300_ immunogenic region-alanine-screening peptides described in Table 1, and PLP_91-110_ (YTTGAVRQIFGDYKTTICGK) were generated by JPT Innovative Peptide Solutions, Berlin Germany. Alanine scanning peptides utilized in the MHC binding assay were biotinylated utilizing an EZ link NHS-Peg4-Biotinylation kit (Thermo Scientific) following the manufacturer’s specifications.

### Animal model

To induce active EAE, 8–12 week old female HLADRB1*03:*01* transgenic mice were anesthetized with tribromoethanol (®Avertin; Sigma Aldrich, St. Louis, MO) 250 mg/kg intraperitoneally (i.p.), and subsequently immunized subcutaneously with proteolipid protein (PLP)_91-110_ (100 μg/100 μl/mouse), hAQP4_281-300_, mAQP4_281-300,_ or whole-length hAQP4 protein (200 μg in 100 μl) emulsified in an equal volume of Complete Freund Adjuvant (CFA) [31] containing 8 mg/mL H37Ra *M*. *Tuberculosis* (Difco, BD, Franklin Lakes, NJ)) in each flank. At the time of immunization and 48 hours later, mice received an i.p. injection of 200 ng pertussis toxin (Ptx) in 200 μL PBS.

For the induction of EAE by adoptive transfer, lymph nodes (LN) of HLADRB1*03:*01* transgenic mice immunized with hAQP4_281-300_ were removed, and single-cell suspensions were prepared. LN cells were cultured in RPMI 1640, supplemented with 5 x 10^−5^ M 2-mercaptoethanol, 2 mM glutamine, 100 μg/ml penicillin, 100 μg/ml streptomycin, 10% fetal calf serum (HyClone, Logan, UT), and stimulated with 25 μgs/ml hAQP4_281-300_ and 0.5 ng/ml IL 12 in a 24 well plate for 72 hours. 5x10^6^ cells per 200 μls phosphate-buffered saline (PBS) were washed with PBS, and naïve HLADRB1*03:*01* mice were subsequently inoculated. Two independent experiments were conducted with a minimum of 3 mice per group for each treatment paradigm.

For all experiments, individual animals were observed daily based on the EAE clinical scoring system as follows: 0 = no clinical disease, 1 = loss of tail tone, 2 = mild paraparesis, 3 = paraplegia, 4 = hindlimb and forelimb paralysis, 5 = moribund or death. Observation of all experimental animals occurred at least twice daily, with documentation of the clinical score. The following interventions are cumulative: Once a mouse reached clinical score 2, moist chow was provided daily. At score 3, animal weights were recorded daily, and animals were euthanized if the weight loss was greater than 20% from baseline. When mice scored 4, the urinary bladder was palpate and manually expressed as needed, and affected animals were no longer housed with cagemates of a lower score. Furthermore, suitable nesting material was always be provided. Affected mice had to be euthanized if there is no improvement after 72 hours at score of 4. Animals were euthanized immediately upon observation of a score of 5, regardless of time to development. Euthanasia was performed by carbon dioxide narcosis. Cervical dislocation was always used as a secondary physical method. Death was confirmed by observing for lack of breathing, loss of heartbeat, glazed appearance to the eyes, and loss of limb movement.

### Histology

Following fixation in 10% buffered formalin, coronal sections of brain tissue, axial sections of spinal cord, and longitudinally-oriented optic nerves were processed and embedded in paraffin blocks. 4 μm sections were cut, mounted on Fisher Brand Superfrost Plus glass slides (Fisher Scientific, Pittsburgh, PA), and stained with hematoxylin & eosin (Fisher Scientific).

For the Luxol Fast Blue stained-sections, 6 μm thick sections of paraffin-embedded tissue were cut on a rotary microtome and mounted on Fisher Brand Superfrost Plus glass slides (Fisher Scientific). The sections were deparaffinized and hydrated. Following heating of the sections in 0.1% Luxol Fast Blue (Sigma-Aldrich, St. Louis, MO) at 60°C for at least one hour, excess stain was rinsed off. They were then differentiated in 70% alcohol for 25 seconds and rinsed in distilled water. Next, the sections were evaluated under the microscopy and depending on the adequacy of differentiation they were subjected to an additional round of lithium carbonate, and an alcohol rinse.

### Pupillometry

The pupillary reflex of experimental mice were measured using the pupillometry system by Neuroptics Inc. (San Clemente, CA) previously described in *Husain et al [32].* Briefly, infrared cameras capture digital images of mouse pupils in darkness at a baseline level after sedation. After the baseline pupil size is determined, an intensity-calibrated light source emits a stimulus into one or both of the eyes and a custom program measures the pupil diameter designed to analyze pupil size, onset latency, constriction velocity, and response amplitude. The light stimulus consists of a flash of light at 2, 32, or 125 μWs.

### T cell proliferation assay

Ten days post immunization of hAQP4_281-300_ or mAQP4_281-300_ of HLADRB1*03:*01* transgenic mice not given Ptx, single cell suspensions were generated by isolating the LNs of the immunized mice. Utilizing the CellTrace Violet Proliferation kit^®^ (Thermo Fisher Scientific, Waltham, MA), CD4^+^ T cell proliferation against antigens was determined. Briefly, isolated 20x10^6^ LN cells were incubated at 37°C for twenty minutes with 5 μM CellTrace Violet in PBS. After incubation, cells were washed with RPMI media twice, then incubated in a 96-well-round bottom plate at 1x10^6^ cells per well with either against hAQP4_281-300_ (5 μg/ml, and 25 μg/mL), mAQP4_281-300_ (5 μg/ml, and 25 μg/mL), media, or ConA (1 μg/mL) then incubated for 96 hours. Post incubation, cells were washed with staining FACS buffer (4% Fetal Calf Serum (FCS) in PBS) two times, then the Fc receptors were blocked with anti-CD16/32 (BD Biosciences, Franklin Lakes, NJ) for 15 minutes at 4°C before staining with mAbs for 30 minutes at 4°C. Cells were stained utilizing the following monoclonal antibodies: CD3-PE-C7 (EBiosciences Cat.#25-0031-82, San Diego, CA), CD4-APC (BD Biosciences Cat#553051), FOXp3-PE (BioLegend Cat#320019, San Diego, CA). A total of 50,000 cells per sample were collected. Data were acquired with a FACS-LSRII (BD Biosciences), and analyzed using FlowJo software (Tree Star, Ashland, OR).

### Tetramer analysis

The frequency of antigen specific CD4^+^ T-cells was assessed using a tetramer binding assay protocol provided by the Benaroya Research institute [33]. Briefly, lymph nodes were harvested, and a single cell suspension was generated. Cells were resuspended in media, and HLADRB1*03:*01* tetramers loaded with hAQP4_284-298_, or empty HLADRB1*03:*01* tetramer (Benaroya Research Institute, Seattle, WA) were added, and incubated for 90 minutes at 37°C and 5%CO_2_. Cells were then washed with FACS buffer and blocked with anti-CD16/CD32, and stained following the protocol for cell surface staining described above with anti-CD3 APC (17A2, Tonbo Biosciences, San Diego, CA) and anti-CD4-Pacific Blue (RM4-5, BD-Biosciences). Data was acquired with a FACS Canto RUO II Special Order (BD Biosciences), and data was analyzed using FlowJo software (Tree Star).

### Enzyme-linked immunosorbent spot assay

The frequency of IFNγ, IL-17, and GM-CSF secreting CD4^+^ T cells were determined by ELISpot assay. Groups of three male HLA-DRB1*03:*01* mice were inoculated in the inguinal and axillary regions with 100 μgs of hAQP4_281-300_ as described above without the addition of Ptx. On day 10, lymph nodes and spleens were collected to generate single cell suspensions. Next, cells (2.5–5.0x10^5^ cells/well) were incubated with a hAQP4_281-300_ (25 μgs/mL), mAQP4_281-300_ (25 μgs/mL), hAQP4_284-299_ (25 μgs/mL), hAQP4_66-79_ (25 μgs/mL), a single hAQP4 alanine peptide (25 μgs/mL), media only, or ConA (1μg/mL) for 48 hours in 96 well ELISpot plates (Millipore MultiScreen 96-Well Plates). Capture and detection of cytokines were accomplished by using monoclonal antibodies (eBiosciences) specific for mouse IFNγ (Clone AN-18 [capture] and R4-6A2 [detection]), IL-17(Clone eBio17CK15A5 [capture] and eBio17B7 [detection]), or GM-CSF (Clone MP1-22E9 [capture] and MP1-2231G6 [detection]). Spots were counted with an automated ELISpot plate reader (Bioreader 5000, Biosys, Karben/Germany).

### MHC binding assay

This assay was adapted from a protocol found in *Busch et al* [34]. Briefly, spleens isolated from naïve HLADRB1*03:*01* transgenic mice were used to generate single cell suspensions. Then, 1x10^6^ splenocytes were incubated with either biotinylated hAQP4_281-300_, mAQP4_281-300_, or hAQP4_284-299_ alanine screening peptides at a concentration of 10μ/mL for 4 hours at 37°C in a 96-well plate. Post incubation, cells were washed two times with FACS buffer and stained for flow cytometry utilizing avdin-FITC (Biolegend) applying the previously described protocol. After staining cells with avidin-FITC, they were run through a Accuri C6 (BD) flow cytometer, or a BD FACSCalibur, and Flowjo was utilized to quantify the percentage of FITC positive cells. A potential delta between the control peptides (hAQP4_284-299_, and mAQP4_281-300_), and the alanine screening peptides is considered to be the result of the alanine substitutions.

### Statistical Analysis

For parametric tests, data were checked for normality by using the Kolmogorov–Smirnov test. Normally distributed values were compared using the unpaired two-sided Student *t*-test. Correlations between continuous and categorical variables were assessed using the Mann-Whitney U-test. All experiments were repeated at least twice. All statistical tests were 2-sided and p < 0.05 indicated significance. All analyses were performed with Prism 5 (Graphpad, La Jolla, CA, USA).
